# Endoscopic filling with polyglycolic acid sheets and fibrin glue for esophagomediastinal fistula after per-oral endoscopic myotomy

**DOI:** 10.1055/a-2749-7695

**Published:** 2025-12-19

**Authors:** Masaki Ominami, Akinari Sawada, Tadashi Ochiai, Daiki Kitagawa, Mitsuhiro Kono, Shusei Fukunaga, Yasuhiro Fujiwara

**Affiliations:** 1 12935 Department of Gastroenterology, Osaka Metropolitan University Graduate School of Medicine, Osaka, Japan

Per-oral endoscopic myotomy (POEM) is an acceptable treatment for achalasia 1 . Fistula formation after POEM for achalasia is a rare but serious adverse event that may require surgical intervention 2 . Polyglycolic acid (PGA) sheets are useful as a conservative treatment for fistula after esophagectomy and delayed perforation after esophageal endoscopic submucosal dissection 3 4 5 .

A 47-year-old woman with achalasia underwent POEM without any adverse intraoperative events. One day after POEM, endoscopy and computed tomography revealed a submucosal hematoma at the esophagogastric junction ( Fig. 1 ). Endoscopy on the 17th day revealed that the submucosal hematoma had disappeared; however, the esophageal mucosa in that area was defective and perforated ( Fig. 2 ). Esophagography using gastrographin confirmed that extraesophageal leakage into the mediastinum, resulting in the formation of an esophagomediastinal fistula ( Fig. 3 ). As endoscopic closure using endoclips appeared difficult, we attempted to repair the fistula by filling it with PGA sheets ( Video 1 ). First, four PGA sheets (Neoveil; Gunze, Tokyo, Japan) cut into 10 × 10 mm sections were held with biopsy forceps and filled into the fistula through the endoscopic channel. A Fibrin glue (Beriplast P Combi-Set; CSL Behring Pharma, Tokyo, Japan) was used to fix the PGA sheets ( Fig. 4 ).

**Fig. 1:**
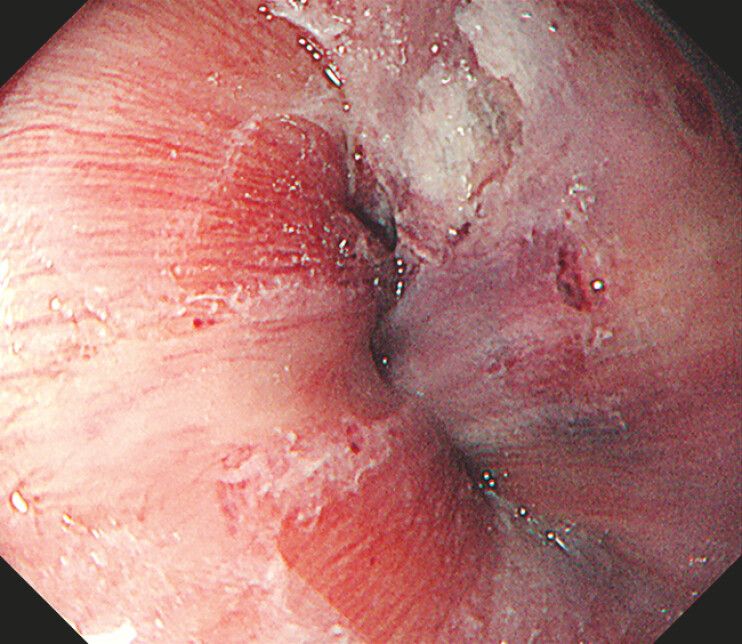
Endoscopic image indicating a submucosal hematoma at the esophagogastric junction after POEM. POEM, per-oral endoscopic myotomy.

**Fig. 2:**
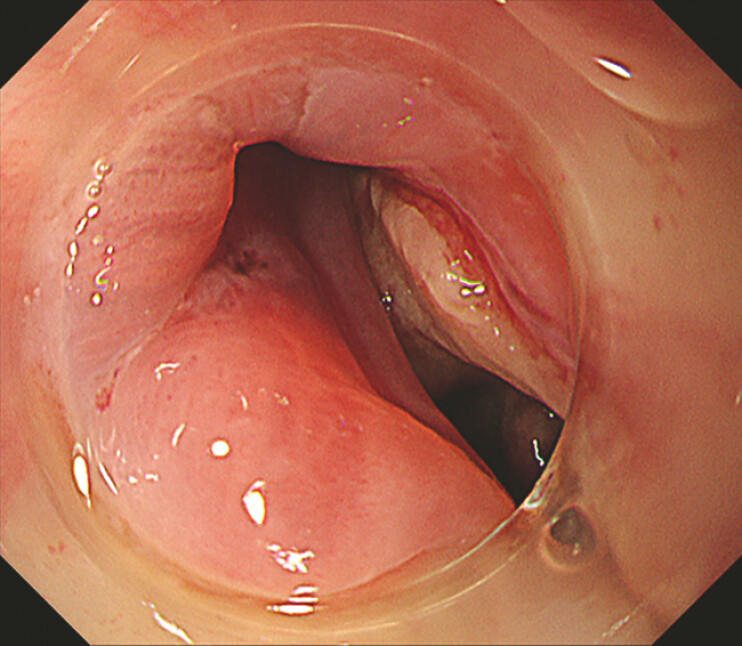
Endoscopic image of perforation and fistula formation at the esophagogastric junction after the hematoma disappeared.

**Fig. 3:**
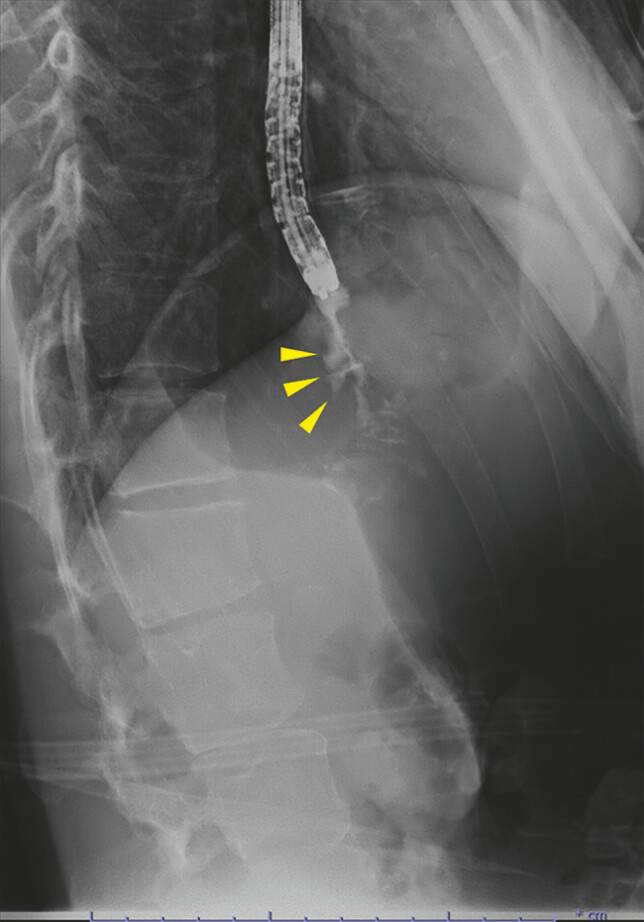
Esophagography using gastrografin revealed an esophagomediastinal fistula (yellow arrowheads).

**Fig. 4:**
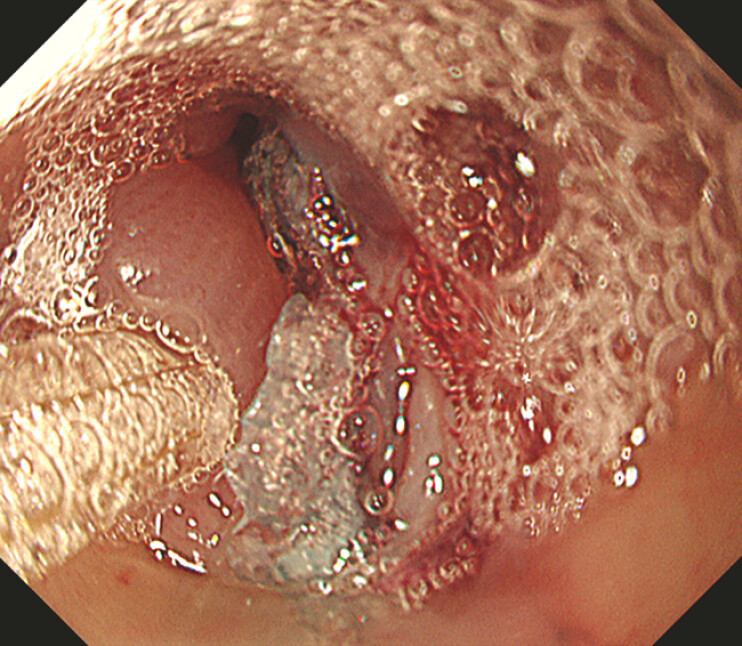
Endoscopic image of a fistula filled with PGA sheets and coated with fibrin glue. PGA, polyglycolic acid.

Endoscopic filling of esophagomediastinal fistulas with PGA sheets and fibrin glue after POEM. POEM, per-oral endoscopic myotomy.Video 1

At 7 and 14 days after filling with PGA sheets, endoscopy revealed that the fistula was filled with regenerated tissue. The absence of leakage outside of the esophagus was confirmed by esophagography with barium. The patient progressed without any problems after starting oral intake, and her dysphagia improved compared to that before POEM. Two months after filling with PGA sheets, endoscopy confirmed that the fistula was closed and that the relaxation dysfunction of the lower esophageal sphincter was improved ( Fig. 5 ).

**Fig. 5:**
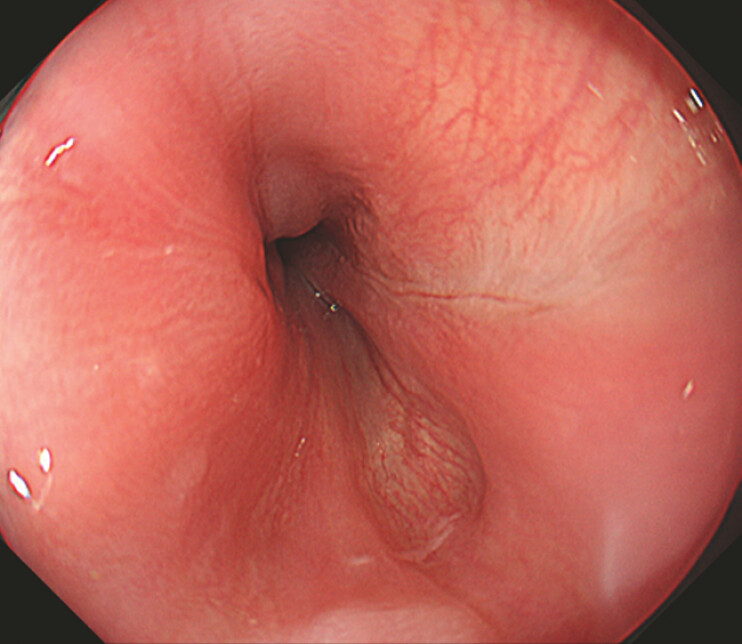
Endoscopic image of the healed fistula at 2 months after filling with PGA sheets. PGA, polyglycolic acid.

Endoscopic filling with PGA sheets and fibrin glue is potentially useful for healing esophagomediastinal fistulas after POEM.

Endoscopy_UCTN_Code_TTT_1AO_2AO

## Notes

Endoscopy E-Videos https://eref.thieme.de/e-videos
*E-Videos* is an open access online section of the journal *Endoscopy* , reporting on interesting cases and new techniques in gastroenterological endoscopy. All papers include a high-quality video and are published with a Creative Commons CC-BY license. Endoscopy E-Videos qualify for HINARI discounts and waivers and eligibility is automatically checked during the submission process. We grant 100% waivers to articles whose corresponding authors are based in Group A countries and 50% waivers to those who are based in Group B countries as classified by Research4Life (see: https://www.research4life.org/access/eligibility/ ). This section has its own submission website at https://mc.manuscriptcentral.com/e-videos .
